# First-Stage Development and Validation of a Web-Based Automated Dietary Modeling Tool: Using Constraint Optimization Techniques to Streamline Food Group and Macronutrient Focused Dietary Prescriptions for Clinical Trials

**DOI:** 10.2196/jmir.5459

**Published:** 2016-07-28

**Authors:** Yasmine Probst, Evan Morrison, Emma Sullivan, Hoa Khanh Dam

**Affiliations:** ^1^ School of Medicine University of Wollongong Wollongong Australia; ^2^ School of Computing and Information Technology University of Wollongong Wollongong Australia

**Keywords:** decision modeling, linear models, dietary requirements, clinical trial, food, programming, linear

## Abstract

**Background:**

Standardizing the background diet of participants during a dietary randomized controlled trial is vital to trial outcomes. For this process, dietary modeling based on food groups and their target servings is employed via a dietary prescription before an intervention, often using a manual process. Partial automation has employed the use of linear programming. Validity of the modeling approach is critical to allow trial outcomes to be translated to practice.

**Objective:**

This paper describes the first-stage development of a tool to automatically perform dietary modeling using food group and macronutrient requirements as a test case. The Dietary Modeling Tool (DMT) was then compared with existing approaches to dietary modeling (manual and partially automated), which were previously available to dietitians working within a dietary intervention trial.

**Methods:**

Constraint optimization techniques were implemented to determine whether nonlinear constraints are best suited to the development of the automated dietary modeling tool using food composition and food consumption data. Dietary models were produced and compared with a manual Microsoft Excel calculator, a partially automated Excel Solver approach, and the automated DMT that was developed.

**Results:**

The web-based DMT was produced using nonlinear constraint optimization, incorporating estimated energy requirement calculations, nutrition guidance systems, and the flexibility to amend food group targets for individuals. Percentage differences between modeling tools revealed similar results for the macronutrients. Polyunsaturated fatty acids and monounsaturated fatty acids showed greater variation between tools (practically equating to a 2-teaspoon difference), although it was not considered clinically significant when the whole diet, as opposed to targeted nutrients or energy requirements, were being addressed.

**Conclusions:**

Automated modeling tools can streamline the modeling process for dietary intervention trials ensuring consistency of the background diets, although appropriate constraints must be used in their development to achieve desired results. The DMT was found to be a valid automated tool producing similar results to tools with less automation. The results of this study suggest interchangeability of the modeling approaches used, although implementation should reflect the requirements of the dietary intervention trial in which it is used.

## Introduction

The measurement of nutrients and prescription of foods for clinical studies can be a difficult task requiring consideration of a number of different elements [1]. When conducting a dietary intervention, it is imperative that researchers ensure consistent allocations of macro- and micronutrients across participants, while also tailoring the required dietary approach to the food requirements, for example, food preferences, of the individual participants as needed. Many high-quality published dietary studies do not report on the use of such tailored approaches and rather apply the underlying assumption that usual dietary intake will be maintained by participants throughout the duration of a trial [2]. However, within a randomized controlled trial design, intervening using a food-/nutrient-based approach will inherently result in changes to dietary intake during the trial, potentially affecting the outcomes. This is particularly evident when a target food is provided to participants, with studies showing that the target food will be eaten in addition to rather than substituted into the usual diet [3], resulting in increased energy (calories or kilojoules) intake due to the intervention. To reduce the effect of these changes, predefined, personalized, energy-focused dietary prescriptions should be implemented via the use of dietary modeling before the intervention.

Modeling is employed to test logic, demonstrate a concept or an idea, and serve as a representation of reality. It often has a mathematical basis [4]. The term “model” also implies variability of the outcomes; hence, multiple options are often tested. In practice, modeling is a theoretical process allowing different scenarios [5] to be created. These scenarios typically stem from an idea, concept, or change of practice. For dietary modeling, the concept generally relates to achieving dietary targets (food or nutrient) by consumption of given amounts (servings) from a range of food groups. It utilizes a combination of food consumption data and/or food composition data [5], although it is not limited to these. In one example of modeling of dietary intake, the concentration of a known nutrient *within* a food is multiplied by the amount of the food consumed to determine the contribution to the total nutrient intake. This type of modeling requires access to food consumption data, such as that of a national survey [6,7]. Modeling conducted by the Australian regulatory authority, Food Standards Australia New Zealand, use tailored software based on the SAS statistical package using the following equation: *dietary intake=Σ(nutrient/chemical concentration × food consumption)* [6]. The software was custom developed to create high-level dietary models at a population level to address chemical exposure and the effect of food contamination [8]. Modeling may also be used to standardize dietary intake across a participant group at the point of intervention such as in a dietary intervention trial, although resource limitations are likely to reduce the opportunity to develop customized software.

The use of tailored tools, specifically for dietary modeling, has the potential to aid the translation from nutrient to food information and incorporate nutrition recommendations [4] related to diet-disease relationships. The tools should ensure a consistent and streamlined process is applied across the entire trial to minimize variability. Manual approaches to prescribing individualized diets are common to dietary studies [9-13]. They are heavily user dependent, providing the potential for a high degree of variability between prescribed outcomes. Manual methods do, however, allow some consideration of the practical issues related to dietary prescriptions at an individual level and food-based guidelines to be incorporated. Practice-based examples include a Microsoft Excel spreadsheet that is manually manipulated to achieve food group targets [14,15] whereby the user, often a dietitian or nutritionist, aims to achieve energy (calories or kilojoules) and macronutrient (total fat, protein, carbohydrate) proportions with minimal variability from the overall trial targets. Modeling is achieved by manually adjusting the number of prescribed servings within a given range (commonly based on dietary guidelines). This process is cumbersome and time consuming for the user and may have significant resource implications within a clinical trial if the dietitian is also needed to monitor the dietary intake of the participants.

Alternatively, adding automation to the modeling process has the potential to decrease the variability and time taken to create the models while still employing a user-dependent approach. The approach utilized to formulate the Australian Dietary Guidelines is an example of modeling with an element of automation, using the Solver Platform for Microsoft Excel [16]. Solver applies a computerized method for finding optimal solutions using predetermined constraints (data limits) to Excel-based spreadsheets. These constraints must be imputed into the tool before it is applied to a spreadsheet. Solver has the potential to minimize the user burden by minimizing inconsistencies from trial targets at a group level but does require extensive input of constraint details. Additionally, Solver does not present the ability to easily optimize across competing objectives that may be found in a clinical trial as it has been designed to primarily provide user-defined constraint satisfaction. The aforementioned spreadsheet-based tools are also limited to food-based output only. They do not consider the need for individualized modeling, a process that would require separate calculation of estimated energy requirements (EER). This individualized approach is common to the highly controlled environment of a dietary intervention clinical trial where each individual needs a targeted dietary prescription rather than a generic one.

The process of dietary modeling in food-based clinical trials when performed manually required dedicated time and resource commitments and the need to calculate energy requirements before development of each model. By applying constraint optimization techniques to this process, it can be automated, saving both time and resources and streamlining the overall approach used. The development of the Dietary Modeling Tool (DMT) [17] has the potential to provide an automated method for dietary prescription, tailored to individual characteristics within the food and/or nutrient-based constraints of a clinical trial. The objectives of the DMT were that it would take on a simple, Web-based (widely accessible), and user-friendly format and target the individual energy requirements of participants. Its development would result in a reduction of between user variability that may become evident when calculating target servings for food groups based on energy requirements using manual and partially automated approaches. Development of streamlined models would also allow users who are not trained in dietetics to create the models, and the dietitians would be employed to address only those models where specific considerations such as food preferences or avoidances need to be addressed. The context for the DMT applied the following assumptions. The clinical trial (study) targets would be generated based on selected food groups to match the nutrition targets defined in the trial. Modeling targets may be specific to the trial or may relate to default nutrition guidelines such as the Dietary Guidelines and Nutrient Reference Values. The clinical trial would have baseline measures for all participants, that is, age, gender, height, and weight (also used to calculate body mass index), available to generate individualized models. This type of demographic information is commonly collected. The overall aim of the tool was that the developed DMT could be easily reconfigured to use multiple macronutrient trial targets; could be adjusted to accommodate a wide range of participant dietary preferences, for example, vegetarian dietary patterns; and could be used across multiple studies, that is, maintain the default preferences for studies A, B, C...X, which may be occurring concurrently. This paper describes the first-stage developmental process of a tool to automatically perform dietary modeling using food group and macronutrient requirements as a test case. The DMT was then compared with existing approaches to dietary modeling (manual and partially automated), which were previously used by dietitians for dietary modeling.

## Methods

The development of the DMT applied lessons from an existing tool developed for the Australian food guidance system (AFGS) that used a *linear* programming approach to modeling. Algorithms published as part of the AFGS were used as the basis for developing the DMT using *nonlinear* modeling. Constraint optimization was also used to ensure the DMT was suited to developing individual dietary prescriptions that are needed in dietary intervention trials as the AFGS targeted population groups. The developed algorithms were combined with existing manual advice models [18] that had been used in published dietary intervention trials conducted by the Smart Foods Centre, University of Wollongong. Second, this paper describes the comparative validation of the DMT against an existing validated manual modeling tool and compares the output with partially automated approaches to dietary modeling. It was hypothesized that the DMT would provide clinically valid results when compared with its manual counterparts while also minimizing user burden.

### The Constraint Optimization Problem

Formally, a constraint is a function CF(Dx_i_,...,Dx_j_) resulting in a Boolean output {True, False}. The result is true if the combination of all values is allowed and false otherwise. An objective function OF(Dx_i_,...,Dx_j_) results in a set of Solutions R, where R is a set of solutions that are optimal with respect to the objectives OF. A constraint optimization problem is a tuple (X,D,C,O) where X is a set {x1,...,xm} of variables, D is a set {d1,...,dm} of variable domains, C is a set {c1,...,cj} of constraints defined over X, and O is a set {o1,...,op} of objective functions defined over X [19].

### Food Guidance Modeling (Using Linear Programming)

Modeling conducted for the AFGS [20] used linear programming [21,22] with population data. Linear programming was used to allow more complex diets to be created within energy and macronutrient restrictions. For example, if a food item or a food group referred to as *i (i=1...m)* is consumed in set amounts shown as *s*_i_ grams (ie, the target serving size), then the daily total intake of a given nutrient *j* is shown by the summation *Σ n*_i_*.s*_i_*.(c*_ij_*/100)*, where *n*_i_ = the consumed number of servings of the food item or food group *i* and *c*_ij_ is the amount of the nutrient *j* per 100 g of the food item or food group *i*. If 100 g of the food item or food group *i* provides *e*_i_ kilojoules of energy to the daily diet, the dietitian then needs to decide on the set of servings *(n*_i_*≥ 0, i=1...m)* from a total list of all possible foods items or food groups *m*, such that the daily total energy intake of *m* is

E=Σ n.s.e

1

and *i=1* is minimized, and such that the guidelines for each of the “nutrients” *j=1...v* are all simultaneously satisfied, that is,

mΣ ni.si.cij/100³ NRVj, j=1...v

Using this approach, it may be necessary to break down the information by food groupings to achieve an outcome.

Further consideration then needs to be given to cultural norms, food preferences, intolerances, palatability, and a number of other factors by applying fixed, a “no more than” or “no less than,” constraints to particular elements of the equation. For example, a maximum intake level might be applied to a food group. At the population level, individual considerations such as food allergies are not addressed. When limitations are applied to the aforementioned process with the aim to minimize energy intake, in order to minimize population obesity levels, a number of solutions may be apparent. These solutions (expressed as numbers) are not always whole integers. As food is generally consumed as a whole item, rounding is required to allow recommendations to be made for a given frequency of consumption, for example, per day or per week. This process results in some minor adjustment to the target energy intake, although this adjustment was found to be negligible [20].

### Dietary Modeling Tool Development

Constraint optimization techniques were used to determine whether nonlinear constraints are better suited to the modeling process when a smaller sample (such as the participants of a dietary randomized controlled trial) is targeted. Constraint optimization is a process whereby a set of rules is created and refined and needs to be upheld for a model to be developed. A constraint optimization problem is a problem that is used to find an optimal assignment of values to a given a set of variables, their domain, a constraint function, and an objective function. A set of variables x1,...,x2 represent values that can be changed; their domain are the acceptable values that each variable can be assigned. For example, given a variable for “servings of vegetables per day” an acceptable domain is 0 to 10. A constraint over the set of variables is a restriction on variables; for example, the energy (calories or kilojoules) of all servings must be less than X. A constraint function is an abstract function that takes a set of variables and their values and returns a True or False answer indicating if they violate the constraints or not. True is returned if all of the constraints are satisfied. A constraint solver is an application that takes in variables and their domain and changes the values of the variables until it can find a satisfactory solution. A satisfactory solution is one where all of the constraints are satisfied and the constraint function returns true. An objective function is a function that takes a set of solutions and determines the best given measure; for example, the objective function “minimize kilojoules” will take a set of results found by the constraint solver and pick the result with the lowest number of kilojoules.

In contrast to the AFGS, it is likely that these constraints will follow a nonlinear form because of the varied considerations needed when modeling a diet, as outlined earlier. The DMT will draw on existing food consumption data collected from published dietary intervention trials as the basis for the weighting of food groups rather than population-focused food consumption data as was applied to AFGS. Output will be provided as the number of servings of key food groups required by a participant, of given energy requirements, to meet the criteria of the clinical trial in which it is being used, optimized against key food group serving suggestions. This is so that a solution diet is not composed of one single food group or presented based on an irregular food group split.

Development of the DMT followed a stepwise process. Food data from completed dietary trials [23-25] were pooled and the percentage contribution of common food groups determined based on macronutrient composition. These food groups were rank ordered under each macronutrient (total fat, protein, carbohydrate) to determine the primary sources. Foods seen to contribute to <75% of the total for each macronutrient were taken as the top foods consumed. Individual foods belonging to the food groupings determined were then categorized by the relative proportions of all macronutrients contained in a single serving. From this the mean energy (kilojoules) and macronutrient content (grams) were determined. Subcategories of food groupings were created based on secondary macronutrients (saturated, monounsaturated, and polyunsaturated fatty acids) and on other nutritive components such as the presence of starch or sugar, which may be required for particular participant groups such as persons with diabetes.

For each food group, the mean nutrient content, standard deviation (SD), range, and coefficient of variation (*CV=SD/mean × 100*) were determined for all foods within that group. Acceptable variation was set at a CV of < 15% for the macronutrients; otherwise, acceptability of the variation was assessed by comparison with an existing food guidance system. Standard deviation and range results were compared with those reported in the 2003 American Diabetes Association Exchange Lists for Meal Planning lists [26]. These lists were considered to be the only comprehensive food exchange lists suited to provide specific data on within-list variations from mean nutrient estimates.

#### Setting Up the Interface

Developed online, the DMT [17] relies on 2 data sources that are not seen by the user to populate the nutrient data (eg, macronutrients) for each of the food groups (eg, vegetables, fruits, and so on):

1. NUTTAB, a reference food composition database [27] for Australia containing a list of all available foods, food groups, their energy, and macro- and micronutrient composition.

2. Dietary intervention trial database containing pooled baseline food intake data from completed trials before intervention.

**Figure 1:**
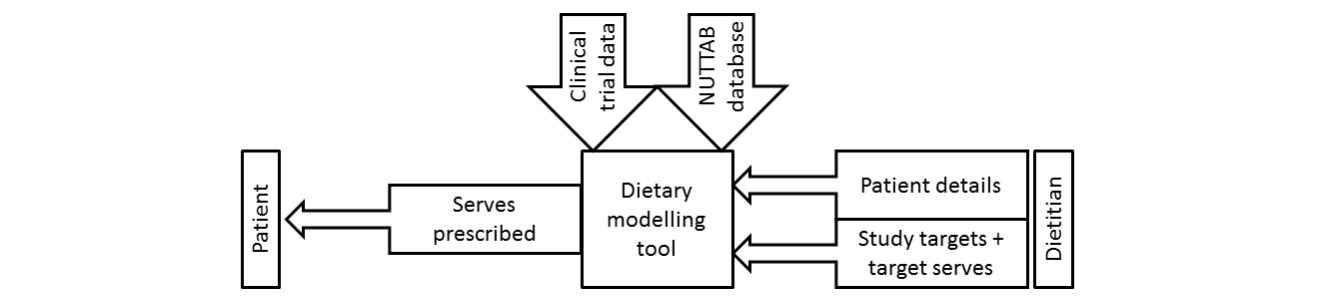
A schematic of the underlying process of using the Dietary Modeling Tool. Note: a dietitian defines the patient details and study targets and target servings per food group and/or per nutrient (study specific), which is entered into the Dietary Modeling Tool.The tool draws data from both a clinical trial and NUTTAB (nutrient tables) database to provide information related to the number of servings suited to the patient details (gender, height, weight, age). These servings are provided to the patient for implementation of the dietary approach.

An overview of the process is shown in Figure 1. In summary, to use the tool, users (likely dietitians) access a website [17]. Initially, the user provides as input to the tool the following data related to the trial:

1. Macronutrient targets for the trial including total fat, protein, and carbohydrate.

2. Target servings *T1, T2, T3,..., Tn* where *Ti* is the target serving for a food group. For example, T1 is the target serving for vegetables (eg, 5 serves), T2 for grains (eg, 6.5), T3 for fruits (eg, 3).

These values are maintained across the trial, and adjustment will be applied to all models created for participants of that trial. Via a separate interface (see Multimedia Appendix 1), the user then inputs the participant details (including height, weight, age, and gender).

The tool then computes the EER from resting energy expenditure (REE) [28] using the following formulae:

For females, REE=9.99×weight+6.25×height–4.92×age–161

For males, REE=9.99×weight+6.25×height–4.92×age+5

EER=REE×PA

Physical activity (PA) is accounted for by standardized activity factors as used in dietetic practice. For the purpose of this first-stage development an activity factor of 1.6 (light activity) was applied [29]. The user may then enter any study-specific macronutrient percentages and desired food group servings (lean meats, dairy, and so on) to suit the participant food preferences. For example, if the participant follows a vegetarian diet the food group servings for meat may be removed by the dietitian and replaced with meat alternatives. This would return to the default trial criteria for the next participant who may not follow a vegetarian diet. The automated DMT would then provide the user with target servings per food group to meet the trial requirements suited to each participant.

#### Creation of the Models

**Figure 2:**
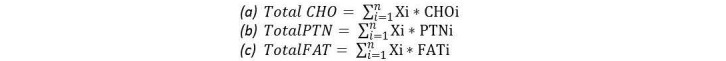
Constraint and objective functions for calculation of food groups based on carbohydrate, protein and fat content.

Then, let *X1, X2,..., Xn* be the servings described for the food groups, for example, X1 for vegetables and X2 for grains. The DMT is needed to determine the “best” value for Xi that meet the constraints and objective functions. Therefore, for carbohydrate (CHO), let TotalCHO be the total of carbohydrates in the servings prescribed shown in Figure 2, equation (a), where *CHOi* is the CHO nutrient data for *Xi.* Similarly, total protein and fat as per equations (b) and (c) in Figure 2 respectively. The resultant total energy of the servings prescribed is then calculated as:

TotalEnergy = TotalCHO + TotalPTN + TotalFAT,

with the percentage energy for macronutrients calculated as given below.

%CHO = [(TotalCHO × 17)/TotalEnergy] × 100

%PTN = [(TotalCHO × 17)/TotalEnergy] × 100

%FAT = [(TotalCHO × 36)/TotalEnergy] × 100

**Figure 3:**
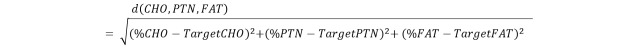
Equation for calculating the Euclidean distance between carbohydrate (CHO), protein (PTN) and fat.

The Euclidean distance between (%CHO, %PTN, and %FAT) and (TargetCHO, TargetPTN, and TargetFAT), referred to as *d(CHO, PTN, FAT)*, was therefore calculated as seen in Figure 3, and, in turn, the tool needs to find the servings prescribed, *Xi*, such that *d(CHO, PTN, FAT)* is minimized, that is, as close to 0 as possible, while the following constraints are satisfied:

1. For every food group i, Xi ≥ Ti (ie, the servings prescribed are greater than or equal to the target serving for every food group).

2. TotalEnergy ≤ EER (ie, the total energy of the servings prescribed is less than or equal to EER).

By example, using the following constraints for prescribed servings the following might be used: X_1_ ≥ 6.5/day (grains), X_2_ ≥ 3/day (fruits), X_3_ ≥ 3/day (dairy).

### Validation and Use in Practice

To evaluate validity, the automated process was compared with a partially automated and manual diet modeling process, and the consistency in servings was compared using the different methods, for a variety of energy levels. As there is no gold standard for validation of dietary models, the manual approach was considered the standard of reference.

For the manual process, a Microsoft Excel calculator [18] used in previous clinical trials conducted at the University of Wollongong was used, and for the partially automated process Microsoft Excel Solver [16] was applied. As all tools were developed based on the same underlying process of food groupings, a consistent food group–based approach could be compared across tools (for vegetables, breads and cereals, fruits, low fat dairy, lean meat, cheese, eggs, oily fish, monounsaturated fatty acid products, and polyunsaturated fatty acid products). Models were created in accordance with the recommendations of the Australian Guide to Healthy Eating [30]. They provided outcomes for the number and size of servings for each food group within the model. Diet models, by food group, were created for 5000 kJ through to 10000 kJ daily intakes, in 500-kJ increments. For comparative purposes the percentage of energy provided from macronutrients was set to be 50% carbohydrate, 20% protein, and 30% fat for all approaches (manual, partially automated, and DMT).

To create comparative data, EER values using sample participant height, weight, age, and gender were calculated using a separate spreadsheet. These data were applied to the Excel calculator and Solver modeling tools. Data for the number of food groups and calculated energy and macronutrient levels were provided by each of the tools.

#### Excel Spreadsheet Calculator (Manual)

An experienced Accredited Practising Dietitian (APD) manually manipulated the number of target servings of food groups in order to achieve appropriate diet models for each kilojoule increment. For repeatability, a less experienced dietitian also performed the same task separately using the Excel spreadsheet calculator [18]. This process created a second comparative set of dietary models for each of the kilojoule targets.

#### Excel Solver (Partially Automated)

The Excel Solver add-in was applied to the aforementioned calculator. In order to determine the appropriate output and ensure consistency, the constraints outlined in Table 1 were applied.

#### Dietary Modeling Tool (Automated)

The sample data for a participant’s height, weight, age, and gender were entered *directly* into the DMT. Trial targets in the study interface were set to the default macronutrient distributions as outlined above.

**Table 1:** Number of serving constraint details per food group applied to the Microsoft Excel Solver modeling tool based on the study by Gillen and Tapsell [19,26].

Food group used for modeling	Number of serving constraint details (per day)	Additional number of serving constraints (required for 8500-10,000 kJ models)
Vegetables	≥ 5, ℤ^a^	
Whole grains	≥ 4, ℤ	
Fruits	≥ 2, <4	
Sugar	≤ 3	Unrestricted
Milk/yoghurt (low/reduced fat)	≥ 2.5	
Milk/yoghurt (whole)	≤ 0	
Soy milk (whole)	≤ 0	
Meat (lean choice, per 30 g)	≥ 3	≥ 5
Cheese (reduced fat, per 30 g)	≥ 0	
Eggs (per 30 g)	≥ 0	
Oily fish	≥ 0.43^b^	
Monounsaturated fatty acids	≥ 0	
Polyunsaturated fatty acids	≥ 0	

^a^ℤ: No upper constraint limit.

^b^ Equates to at least 1 serving per week.

### Data Analysis

The variability of the percentage of energy from each of the macronutrient targets was calculated for all tools. The outcomes from each of the dietary modeling approaches were compared for grouped food data for each of the kilojoule increments tested. The percentage difference of each of the methods, in comparison with the reference method (output from the manual process created by an experienced dietitian), was calculated in order to determine the comparative validity of the processes.

## Results

Constraint optimization was found to be a suitable approach to tool development. As outlined earlier, the DMT was developed with 2 interfaces for users: a study interface, for defining default constraints for a study, and a user interface for modeling individual participant diets within the selected trial. Screenshots of these user screens are provided in Multimedia Appendix 1 [17]. This approach to development allows multiple users to model diets in different studies simultaneously. The forms were hosted online [17] also allowing multi-user access from varied locations.

**Table 2:** Target servings prescribed for each food group using the manual, partially automated, and automated approaches applied to different energy frameworks.

No.	Model^a^ (kJ^b^ target)	Vegetables (%∆)	Grains (%∆)	Fruits (%∆)	Dairy (%∆)	Lean meat, 30 g (%∆)	Cheese^c^, 30 g (%∆)	Eggs^d^, 1 egg (%∆)	Fish^e^, 90 g (%∆)
1.	Reference (5000)	5.00	5.00	2.00	2.00	3.00	0.29	0.58	0.43
	Partially automated	5.00 (0)	4.00 (20)	2.00 (0)	2.50 (25)	3.00 (0)	0.00 (100)	0.00 (100)	0.43 (0)
	DMT^f^	5.00 (0)	4.50 (10)	2.00 (0)	2.00 (0.)	3.00 (0)	0.00 (100)	0.00 (100)	0.00 (100)
	Manual	5.00 (0)	5.00 (0)	2.00 (0)	2.50 (25)	3.00 (0)	0.00 (100)	0.29 (51)	0.43 (0)
2.	Reference (5500)	5.00	5.00	2.00	2.00	3.50	0.29	0.58	0.43
	Partially automated	5.00 (0)	4.00 (20)	2.30 (15)	2.50 (25)	3.00 (14)	0.00 (100)	0.00 (100)	0.43 (0)
	DMT	6.00 (20)	7.00 (40)	2.07 (4)	2.00 (0)	3.00 (14)	0.00 (100)	0.00 (100)	0.00 (100)
	Manual	5.00 (0)	5.00 (0)	2.00 (0)	2.50 (25)	3.00 (14)	0.00 (100)	0.29 (51)	0.43 (0)
3.	Reference (6000)	5.00	6.00	2.00	2.00	3.50	0.29	0.58	0.43
	Partially automated	5.00 (0)	4.00 (33)	2.99 (50.)	2.50 (25)	3.00 (14)	0.00 (100)	0.00 (100)	0.43 (0)
	DMT	6.44 (29)	7.00 (17)	2.50 (25)	2.17 (9)	3.00 (14)	0.00 (100)	0.00 (100)	0.12 (73)
	Manual	5.00 (0)	6.00 (0)	2.00 (0)	2.50 (25)	3.50 (0)	0.00 (100)	0.29 (51)	0.43 (0)
4.	Reference (6500)	5.00	6.00	2.00	3.00	4.00	0.29	0.58	0.43
	Partially automated	5.00 (0)	4.21 (30)	4.00 (100)	2.50 (17)	3.00 (25)	0.00 (100)	0.00 (100)	0.43 (0)
	DMT	7.00 (40)	7.38 (23)	2.50 (25)	2.49 (17)	3.20 (20)	0.09 (69)	0.03 (96)	0.43 (0)
	Manual	5.00 (0)	6.00 (0)	2.00 (0)	2.50 (17)	3.50 (13)	0.29 (0)	0.29 (51)	0.43 (0)
5.	Reference (7000)	5.00	6.00	2.00	3.00	4.00	0.29	0.58	0.43
	Partially automated	5.00 (0)	5.21 (13)	4.00 (100)	2.53 (16)	3.31 (17)	0.09 (69)	0.16 (72)	0.18 (58)
	DMT	7.00 (40)	9.25 (54)	2.50 (25)	2.51 (16)	3.00 (25)	0.04 (85)	0.00 (99)	0.17 (60)
	Manual	5.00 (0)	6.00 (0)	3.00 (50)	2.50 (17)	3.50 (13)	0.29 (0)	0.29 (51)	0.43 (0)
6.	Reference (7500)	5.00	7.00	2.00	3.00	4.00	0.29	0.58	0.86
	Partially automated	5.00 (0)	5.78 (17)	4.00 (100)	3.00 (0)	3.04 (24)	0.00 (100)	0.00 (100)	0.43 (50)
	DMT	7.00 (40)	9.25 (32)	4.00 (100)	3.07 (2)	3.00 (25)	0.00 (100)	0.01 (99)	0.86 (0)
	Manual	5.00 (0)	7.00 (0)	3.00 (50)	2.50 (17)	4.00 (0)	0.00 (100)	0.29 (51)	0.43 (50)
7.	Reference (8000)	5.00	8.00	3.00	3.00	4.50	0.29	0.86	0.86
	Partially automated	5.00 (0)	6.27 (22)	4.00 (33)	3.00 (0)	4.50 (0)	0.00 (100)	0.00 (100)	0.43 (50)
	DMT	7.00 (40)	9.25 (16)	4.00 (33)	4.00 (3)	3.00 (33)	0.26 (10)	0.00 (100)	0.75 (13)
	Manual	5.00 (0)	7.00 (13)	3.00 (0)	2.50 (17)	5.00 (11)	0.29 (0)	0.58 (33)	0.43 (50)
8.	Reference (8500)	5.00	8.00	3.00	3.00	5.00	0.43	0.86	0.86
	Partially automated	5.00 (0)	7.34 (8)	4.00 (33)	3.00 (0)	5.00 (0)	0.00 (100)	0.00 (100)	0.43 (50)
	DMT	7.00 (40)	9.25 (16)	4.00 (33)	4.00 (33)	3.32 (34)	0.26 (40)	0.00 (100)	0.43 (50)
	Manual	5.00 (0)	7.50 (6)	3.00 (0)	3.00 (0)	5.00 (0)	0.29 (33)	0.58 (33)	0.43 (50)
9.	Reference (9000)	5.00	9.00	3.00	3.00	5.00	0.29	0.86	0.86
	Partially automated	5.00 (0)	8.12 (10)	4.00 (33)	3.00 (0)	5.00 (0)	0.00 (100)	0.04 (95)	0.58 (33)
	DMT	7.00 (40)	9.25 (3)	4.00 (33)	4.00 (33)	5.00 (0)	0.29 (0)	0.17 (81)	0.50 (42)
	Manual	5.00 (0)	8.00 (11)	3.50 (17)	3.00 (0)	5.00 (0)	0.43 (50)	0.58 (33)	0.86 (0)
10.	Reference (9500)	5.00	9.00	4.00	3.00	6.00	0.43	0.86	0.86
	Partially automated	5.00 (0)	9.01 (0)	4.00 (0)	3.00 (0)	5.23 (13)	0.00 (100)	0.18 (79)	0.58 (33)
	DMT	7.00 (40)	9.25 (3)	4.00 (0)	4.00 (33)	5.00 (17)	0.29 (33)	0.57 (33)	0.50 (42)
	Manual	5.00 (0)	8.50 (6)	4.00 (0)	3.00 (0)	5.00 (17)	0.43 (0)	0.58 (32)	0.86 (0)
11.	Reference (10,000)	5.00	9.00	4.00	3.00	6.00	0.43	1.43	0.86
	Partially automated	5.00 (0)	10.07 (12)	4.00 (0)	3.00 (0)	5.45 (9)	0.05 (88)	0.15 (90)	0.55 (36)
	DMT	7.00 (40)	9.25 (3)	4.00 (0)	4.00 (33)	5.00 (17)	0.29 (33)	0.57 (60)	0.50 (42)
	Manual	5.00 (0)	8.50 (6)	4.00 (0)	3.00 (0)	6.00 (0)	0.43 (0)	0.86 (40)	0.86 (0)

^a^ Reference method employed was the use of a manual spreadsheet-based tool used by an Accredited Practising Dietitian. Partially automated process applied Microsoft Excel Solver application.

^b^ kJ: kilojoule.

^c^ 0.14=1/week, 0.286=2/week.

^d^ 0.286=1/week, 0.58=2/week, 0.86=3/week.

^e^ 0.43=1/week, 0.86=2/week.

^f^ DMT: Dietary Modeling Tool.

When data were compared with the other modeling forms, the general trend for the output across the 4 dietary models in each kilojoule increment was relatively similar (Table 2). The greatest consistency was seen between the 2 manual approaches. Notably, for a considerable number of food groups across the kilojoule targets, there was no difference between these 2 models, justifying use of the manual process as the standard of reference due to repeatability of the data. The DMT outcomes were the most varied from those created using the reference process. The outcomes for monounsaturated fatty acids and polyunsaturated fatty acids (subnutrients) across the modeling tools were highly variable, with differences of up to 200% for the 5000-kJ and 5500-kJ targets, data not shown. In this instance the reference model was prescribing 1 teaspoon monounsaturated fatty acids, as opposed to 3 teaspoons from the DMT. Less variation was evident for these particular food groups in the higher kilojoule targets (≥8000 kJ). The lean meat prescription was largely consistent across all of the dietary models. The largest difference in the lean meat outcomes was seen for the 8000-kJ and 8500-kJ targets, with 33% and 34% differences, respectively, between the DMT and reference model. This difference equated to one and a half servings (one serving = 30 g) of lean meats. Furthermore, the prescription of fish from the 5000-kJ to the 7000-kJ targets for the reference method and solver models all equated to 90-g servings of fish per week. The DMT produced the same results for the 6500-kJ target; however, for kilojoule targets between 5000 kJ and 7000 kJ it prescribed either much less or no oily fish for the dietary models. Furthermore, there was little difference in the prescription of vegetables for each of the kilojoule targets, although the DMT prescribed up to 2 extra servings (up to 1 cup extra) than the other models.

## Discussion

### Principal Findings

The study described in this paper addressed the first-stage development of and comparative validation of an automated DMT. Applying lessons from previous linear modeling work, a dietary modeling tool using constraint optimization and nonlinear programming was developed online incorporating the expected energy requirement calculations in the same system rather than as separate data as per other less automated tools. Having the DMT online has the potential for improved user access, and creating an algorithm with constraints applied to it should also minimize the variability by comparison with manual modeling approaches. The identified differences between the approaches were not found to be clinically relevant. Although it was not the focus of this paper, it is likely that improved time efficiencies were created by automation of the DMT because of the incorporation of the EER calculation into the user interface. Furthermore, a reduced need for “guess work,” as is common to manual approaches, is also a significant advantage.

When comparing the dietary models created by the APD as the reference for comparison, and the models created by the less experienced dietitian student (manual models), the results across each of the kilojoule increments were most similar. It is likely that because of their training both users were aware of practical servings of particular food groups. Resultantly, excessive or limited servings of certain food groups were not identified in these 2 methods. Furthermore, being trained in the field of nutrition and dietetics and working closely with the Australian Dietary Guidelines, the diet models created by both users were most consistent with these recommendations. The partially automated models produced the next most similar outcomes to the reference models, although they still produced acceptable output for the majority of the kilojoule increments based on the Australian Dietary Guidelines. A benefit of using the Solver add-on was the restrictions that were able to be placed on the variability of energy coming from the macronutrients, ensuring the maximum reliability of individualized diets within a trial. Where possible, these were set within 3 percentage points of the macronutrient targets specified across each of the tools (50% carbohydrate, 20% protein, and 30% total fat). The DMT was also able to suggest appropriate dietary models for the majority of the kilojoule increments within these set limits. This was particularly evident for the higher kilojoule targets. The reference model and manual approach were not able to maintain such limited variability. The variability of the percentage of energy from macronutrients in the DMT fluctuated. For the majority of the models the variation between macronutrient contribution and the target was smaller than that seen in both the reference and manual models. The DMT output demonstrated the greater variation from the reference models; however, it must be noted that models could not be created based on the exact kilojoule increment required for comparison as the other 3 models, as the DMT results were created based on a subject’s *exact* EER rather than rounded to the nearest 500-kJ increment, demonstrating that it could be tailored to each individual participant of a clinical trial. The manual and partially automated methods were not tailored specifically to each individual because of the time-consuming nature of obtaining the modeling outcomes. Therefore, the outcomes gathered were closest to the required kilojoule increments. For a dietary intervention trial, diet models that have been specifically designed to meet the individual requirements of a participant are more desirable and, consequently, the method employed by the DMT would be preferable over the comparative methods in this study.

The largest variation across the dietary models was seen first, for the polyunsaturated fatty acids and second, for the monounsaturated fatty acids food groups. Interestingly, there is no exact recommendation in the Australian Dietary Guidelines in terms of servings related to these nutrients that could be referenced to create the models. It can be seen that these fats were largely used to make up the difference in energy and percentage of energy from fats required once all other food groups had been assigned, potentially explaining the difference. They also flag the need for a modification to the algorithms when nutrients beyond the macronutrient level are to be considered.

### Limitations

Challenges arose with the partially automated method and the DMT in terms of gaining appropriate outcomes. The Solver had to be tested with a range of constraints until desirable outcomes were being achieved across the kilojoule increments, again taking additional time until the desired result was achieved. When too many restrictive constraints were added, the tool was unable to compute a result. Therefore certain limitations (particularly leniency with the degree of variability from the set macronutrient targets) had to be made more liberal. The dietary modeling framework described in this paper had a number of developmental challenges to be overcome in order to produce comparative data. This included having the same results produced for each participant, irrespective of significant differences in age, height, and weight. Second, the outcomes produced were excessive in certain food groups (prescribing up to 7 servings of fruit daily) and limited in others (food groups contributing fatty acids, which were adjusted to be more liberal). Each challenge was overcome by modifying the constraints of the algorithm. Furthermore, serving sizes were altered to those traditionally recommended in dietetics, such as half a cup of cooked vegetables and 30 g of cheese for a serving, to make the tool more practical for use in food-based trials rather than based directly on the Excel calculator, which was developed based on exchange lists. A final alteration required the number servings of food groups to change depending on the EER of the participant rather than manipulating the frequency (in hours) that a food group should be consumed. The latter method assumed that individuals eat continuously over a 24-hour period, a case where professional judgment was needed to adjust the algorithm. Again, overcoming these issues when validating the DMT has ensured it is much more practical and simple to use and demonstrated that it can produce dietary models for macronutrient-based targets. It does, however, emphasize the importance of a nutrition expert, using the professional judgment [31], and working alongside computer programmers when developing tools for use in practice. Some limitations that could not be overcome within the current project include the inclusion of key nutrients beyond macronutrients. Inclusion of key nutrients in DMT, beyond the current macronutrients, will allow for a wider application to practice. The current form limits the use of the tool to studies related to overall energy constraints such as weight management or diabetes. Studies that aim to control dietary intake of vitamins and minerals could not use the tool in its present form. Furthermore, there is still a degree of professional judgment required with regard to the development of diet models. Complete individualization for food allergies or intolerance is not possible, although the researchers believe care professional judgment of appropriately qualified professionals such as dietitians should still be maintained in this instance.

### Comparison With Prior Work

It was also found that despite the type of modeling used in this study, careful consideration needed to be given to the rounding up or down of target servings. This was also identified in the AFGS [20] with rounding found to have minimal effect on the total energy being recommended. For the models of this study, models were largely rounded up to the nearest whole integer, although an underlying assumption was held that the final values would be used to create practical advice. Furthermore, the translation of food information from numbers determined in a model to practical suggestions provided by a dietitian in practice can vary significantly. This was also noted in the AFGS models as an additional consideration that needs to be addressed when liaising with an individual. Food allergies, food intolerances, and food preferences are only 3 of these considerations and are separate from those such as economic shifts, which may affect willingness to buy or purchase particular meat cuts, for example, or sustainable produce in relation to fish intake. Although DMT was developed to minimally address food preferences at a food group level, considerations in addition to this do require professional input and may not be appropriate to automate. Limitations were also apparent in comparing the data as certain food groups varied across the modeling methods. The DMT grouped fish with the other meats and protein-containing foods as is evident in the Australian Dietary Guidelines, instead of being a separate group as the other models. For the purpose of this study, to translate the information into practical suggestions for a participant and for the comparison to be made, these foods were separated by the dietitian into an appropriate amount of lean meats (based on what was prescribed by the tool) and the remaining portion considered as the fish.

### Conclusions

Dietary modeling is essential for the formulation of food-based prescriptions and useful to standardize background diets within randomized controlled trials. In its present form DMT provides this by using predefined macronutrient proportions for all participants of a dietary trial at the point of intervention. As demonstrated in this study, different dietary modeling tools with the same dietary targets produced similar results. Manual methods for dietary modeling are less ambiguous in terms of the desired outcomes, as the model creator is aware of quantity prescriptions classified as being “appropriate.” This method, however, requires trained dietary professionals to be able to produce desired results. When creating individualized prescriptions this can be a time-consuming process. Partially or fully automated methods such as the use of Solver and the developed DMT have the potential to be practically applicable for widespread use in dietary research. The DMT was found to be a valid automated tool producing similar results to tools with less automation. Once the underlying constraint systems have been formulated appropriately, use of such tools may not require trained professionals or those familiar with the Australian Dietary Guidelines for the development of all models, saving this expertise for practical translation of the models. This has significant resource implication for a research trial and even more so when considering the time saved when compared with the manual approaches used for dietary modeling. Future refinements are necessary to consider other nutrients such as key vitamins and minerals in the models to increase the flexibility of the tool and widen its application to practice. Further to this, inclusion of prompts within DMT related to food allergies or intolerances could also be included for further refinement of the model with a qualified practitioner.

Partially automated approaches such as that of Solver still require increased time to set the constraints; however, they will not produce results that are practically viable. Although by comparison with the manual method Solver does save some time, it continues to require the input of a professional to ensure the models produced are realistic. It is observed that although time was not monitored as part of this study, increased automation appears to relate to an increase in time saved and may in turn result in a reduction in resource allocations to the trial. With respect to the DMT the results of this study suggest interchangeability of the approaches, although implementation should reflect the requirements of the trial in which it is used and the available resources that can be used.

## Appendix

Multimedia Appendix 1 Screen shots of the Dietary Modelling tool showing the study targets, patient details and food group output screen available from http://dietmodels.com/dmt.

## Abbreviations

AFGS: Australian food guidance system
APD: Accredited Practising Dietitian
CHO: Carbohydrate
DMT: Dietary Modeling Tool
EER: estimated energy requirement
NRV: Nutrient Reference Value
PA: physical activity
REE: resting energy expenditure
