# Supplementary material for: First-Stage Development and Validation of a Web-Based Automated Dietary Modeling Tool: Using Constraint Optimization Techniques to Streamline Food Group and Macronutrient Focused Dietary Prescriptions for Clinical Trials
Source: J Med Internet Res. 2016 Jul 28;18(7):e190. doi: 10.2196/jmir.5459 (PMC4981694; doi:10.2196/jmir.5459)
Supplement: Multimedia Appendix 1 [file jmir_v18i7e190_app1.pdf]

Supplementary material: Screen shots of the Dietary Modelling tool showing the study targets, patient details and food group output screen available from http://dietmodels.com/dmt

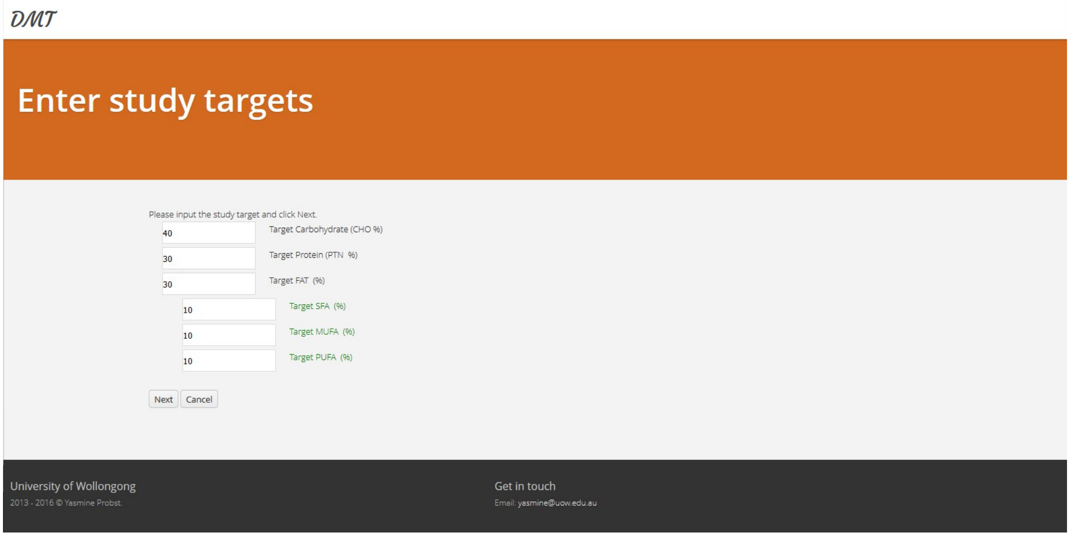

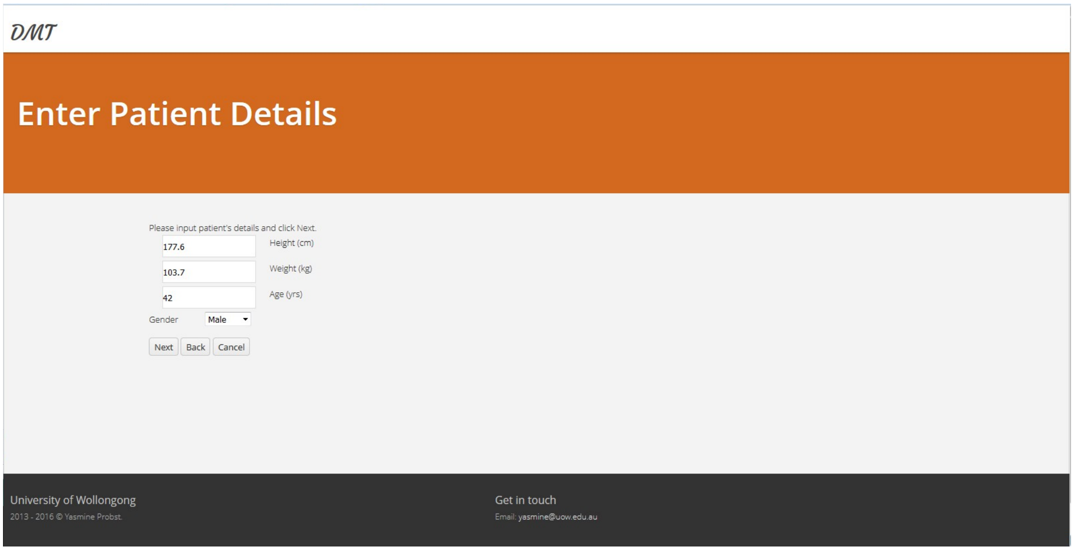

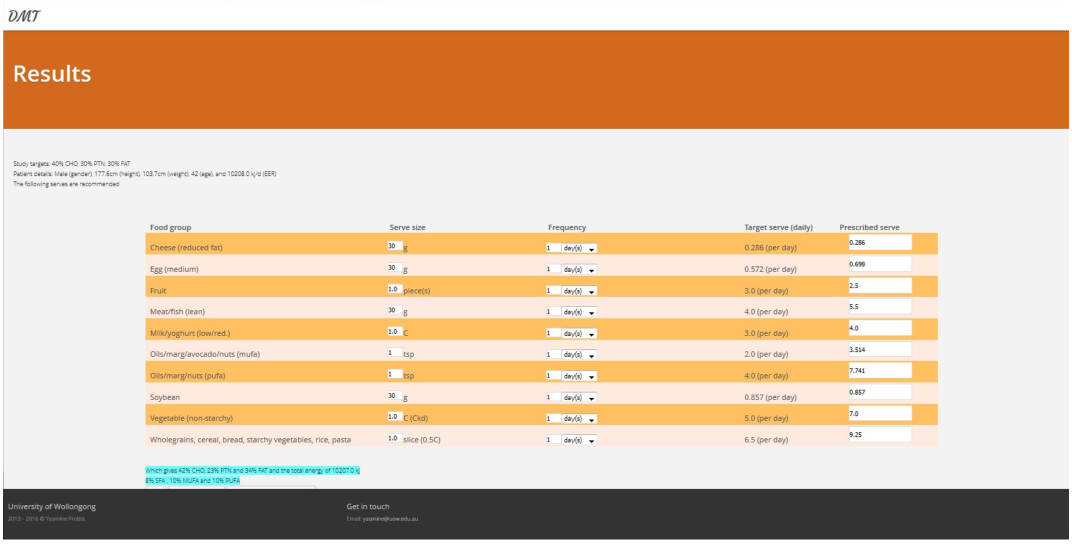

Food group	Serve size	Frequency	Target serve (daily)	Prescribed serve
Cheese (reduced fat)	30 g	1 day(s)	0.286 (per day)	0.286
Egg (medium)	30 g	1 day(s)	0.572 (per day)	0.698
Fruit	1.0 piece(s)	1 day(s)	3.0 (per day)	2.5
Meat/fish (lean)	30 g	1 day(s)	4.0 (per day)	5.5
Milk/yoghurt (low/red.)	1.0 C	1 day(s)	3.0 (per day)	4.0
Oils/marg/avocado/nuts (mufa)	1 tsp	1 day(s)	2.0 (per day)	3.514
Oils/marg/nuts (pufa)	1 tsp	1 day(s)	4.0 (per day)	7.741
Soybean	30 g	1 day(s)	0.857 (per day)	0.857
Vegetable (non-starchy)	1.0 C (Ckd)	1 day(s)	5.0 (per day)	7.0
Wholegrains, cereal, bread, starchy vegetables, rice, pasta	1.0 slice (0.5C)	1 day(s)	6.5 (per day)	9.25
